# First-Principles Study on the Photocatalytic Performance of K(Ta_0.5_Nb_0.5_)O_3_ Doped with Metals (Cd, Sn, Hf)

**DOI:** 10.3390/nano15171322

**Published:** 2025-08-28

**Authors:** Can Zhao, Qiao-Yue Chen, Xin-Yuan Zhou, Xu-Cai Zhao, Bo-Cheng Lei, Li-Li Zhang, Jing Zhao, Yi-Neng Huang

**Affiliations:** 1 Xinjiang Laboratory of Phase Transitions and Microstructures in Condensed Matters, College of Physical Science and Technology, Yili Normal University, Yining 835000, China; zhaocan@ylnu.edu.cn (C.Z.); chenqiaoyue@ylnu.edu.cn (Q.-Y.C.); zhouxinyuan@ylnu.edu.cn (X.-Y.Z.); 21081@ylnu.edu.cn (X.-C.Z.); leibocheng@ylnu.edu.cn (B.-C.L.); 2 Xinjiang Key Laboratory of Clean Conversion and High Value Utilization of Biomass Resources, School of Chemistry and Chemical Engineering, Yili Normal University, Yining 835000, China; 13016@ylnu.edu.cn; 3 National Laboratory of Solid State Microstructures, School of Physics, Nanjing University, Nanjing 210093, China

**Keywords:** optical properties, effective mass, K(Ta_0.5_Nb_0.5_)O_3_, first principle

## Abstract

Based on the excellent performance of the K(Ta_0.5_Nb_0.5_)O_3_ (KTN) system, this study systematically investigated the mechanism of the influence of metal element (Cd, Sn, Hf) doping on the photocatalytic performance of KTN ferroelectric materials using the density functional theory (DFT) based on first principles. The findings indicate that after metal atom doping, the tolerance factor of doping systems is similar to that of pure KTN crystals, confirming that doping does not compromise its structural stability. However, the ion radius differences caused by doping lead to lattice distortion, significantly reducing the bandgap width. Because the impurity element substituting the Ta site exhibits a lower valence state compared to Ta, holes become the majority carriers, thereby endowing the semiconductor with p-type characteristics. These characteristics effectively suppress electron–hole recombination while enhancing electron transitions. Furthermore, the increase in the dielectric constant of the doped system indicates an enhancement in its polarization capability, which is accompanied by a significant improvement in carrier mobility. The peak of the imaginary part of the dielectric function and the peak of the absorption spectrum both shift towards the low-energy region, indicating that doping has expanded the light response range of the system. Moreover, the effective mass of the holes in all doped systems is significantly higher than that of the electrons, further demonstrating that the introduction of impurities is conducive to hindering the separation of photogenerated electron–hole pairs. These modifications significantly enhance the photocatalytic performance of the systems.

## 1. Introduction

With rapid socio-economic development and a sharp increase in population, environmental pollution and energy shortages have become increasingly severe, posing serious threats to human survival. In this context, photocatalytic technology presents a promising solution to these pressing challenges. This technology employs light-responsive materials that absorb solar energy and generate electron–hole pairs, which subsequently drive redox reactions. In 1972, Fujishima and Honda [1] first reported that titanium dioxide (TiO_2_) could decompose water into hydrogen and oxygen under ultraviolet (UV) light irradiation, sparking a research boom in photocatalytic materials [2,3,4]. Over the following decades, traditional semiconductor photocatalysts analogous to TiO_2_, such as SnO_2_, ZrO_2_, CdS, WO_3_, and Fe_2_O_3_, were successively developed. However, these materials typically exhibit wide band gaps and a high recombination rate of photogenerated carriers (electron–hole pairs) [4], leading to difficulties in enhancing solar energy conversion efficiency. Therefore, exploring novel high-performance semiconductor photocatalytic materials is crucial.

Grosso et al. [5] pioneered the application of ferroelectric materials in photocatalysis in 2004. Since then, ferroelectric materials have garnered widespread attention due to their unique spontaneous polarization properties, particularly in the field of emerging photocatalysts [6,7]. The internally self-generated polarization electric field can effectively suppress the recombination of photogenerated electron–hole pairs [8], thereby overcoming the critical limitation of traditional semiconductor photocatalysts in terms of carrier recombination efficiency. Within these materials, KTN has risen to prominence as a key research focus due to its outstanding thermal, chemical, and mechanical stability [9,10]. The KTN crystal features a cubic lattice structure, with potassium (K) atoms occupying the vertices, oxygen (O) atoms located at the face centers, and tantalum (Ta) and niobium (Nb) atoms co-occupying the body center position. By adjusting the Ta/Nb ratio, the crystal can form complex ferroelectric domain structures. Disparate ferroelectric domains within the crystal undergo reconstruction driven by energy competition, forming a three-dimensional (3D) ferroelectric supercell with spatially distributed polarization. This unique structure effectively facilitates the rapid separation and transport of photogenerated electrons and holes within the crystal, significantly reducing charge carrier recombination and thereby enhancing photocatalytic performance [11]. Leveraging these properties, KTN crystals exhibit promising potential in photocatalytic applications. For instance, diamond films grown on KTN substrates demonstrate exceptional photocatalytic performance, achieving degradation efficiencies for rhodamine B dye as high as 98% within 10 h under 365 nm UV irradiation [12]. Although the spontaneous polarization intensity of the KTN system is lower than that of K_0.5_Na_0.5_NbO_3_ (KNN) and NaNbO_3_ (NN) materials, it exhibits the best thermodynamic stability [13]. This enables the effective dissociation of photogenerated carriers in KTN, resulting in superior photocatalytic activity. The spontaneous polarization coefficients and Curie temperature data of these systems are shown in Table S4 of Supplementary Materials.

However, KTN materials possess a relatively large bandgap (~3.29 eV), requiring electrons within the system to overcome a higher energy barrier during excitation [14]. Consequently, the photocatalytic efficiency of KTN still requires further enhancement. Researchers widely employ doping modification strategies to improve the photocatalytic performance of ferroelectric materials. For instance, studies demonstrated that doping ZrCoH_3_ systems with hafnium triggers lattice distortion, dramatically expanding the materials’ visible light absorption spectrum [15]. This approach enhanced the catalytic efficiency of the systems to 38.23%, demonstrating a substantial improvement in photocatalytic activity. Research by Cui Zongyang et al. shows that Sn-doped BiFeO_3_ can regulate the band structure, enhance visible light absorption and carrier migration efficiency [16]. Similarly, Zhang Jie et al. [17] found that doping potassium sodium niobate (KNN) systems with Ni and Sr elements effectively reduced their optical bandgap and enhanced visible light absorption capacity, thereby significantly boosting the photocatalytic performance of KNN. Furthermore, Xu Yuan et al. observed that Cd^2+^ doping significantly improved the defect state density of CsPbIBr_2_ perovskite materials, suppressed photogenerated carrier recombination, achieved energy-level optimization, and enhanced light conversion efficiency [18]. These studies clearly demonstrate that metal element doping significantly improves the photocatalytic performance of ferroelectric materials, establishing it as a viable modification approach. This evidence also inspires the reasonable hypothesis that metal doping may effectively enhance the photocatalytic performance of the KTN ferroelectric system.

Nevertheless, research on enhancing the photocatalytic activity of KTN via metal doping has been relatively scarce. To gain deeper insights into the photocatalytic mechanisms of KTN ferroelectric materials, this study systematically investigates the effects of doping KTN with transition metal elements (Cd, Sn, Hf). For the convenience of comparison, fifth- and sixth-period transition metals Cd, Sn, and Hf, with similar electronic structures, were selected as doping atoms. The selected metals possess stable crystal structures at room temperature and readily form compounds with various elements, making them suitable dopants. Among them, the valence electrons of Cd and Hf primarily occupy d orbitals, whereas those of Sn are mainly distributed in p orbitals. This diversity in electronic structure enables these metals to effectively modulate the band structure of the photocatalysts and suppress electron–hole recombination [19,20]. This work will conduct an in-depth analysis of changes in the crystal structure, electronic structure, optical properties, and carrier effective mass of the KTN system before and after doping. The aim is to develop material systems with higher photocatalytic efficiency and provide a reliable theoretical foundation for subsequent experimental research.

## 2. Materials and Methods

**Figure 1:**
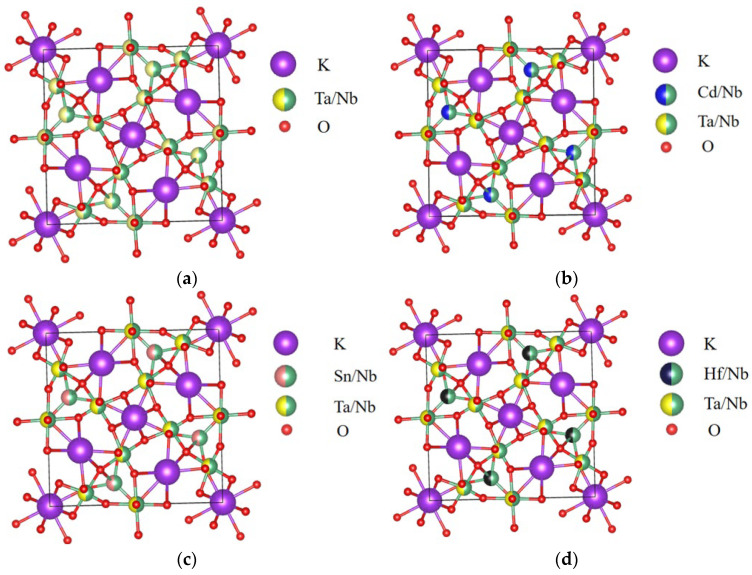
Model diagrams of the KTN systems before and after doping: (**a**) KTN; (**b**) KTN-Cd; (**c**) KTN-Sn; (**d**) KTN-Hf.

This paper investigates the KTN solid solution with a 2 × 2 × 2 tetragonal structure formed by 50% potassium niobate (KNbO_3_, KN) and 50% potassium tantalate (KTaO_3_, KT), and the space group is P4mm(No. 99) [21], with lattice constants a = 8.0248 Å, b = 8.0248 Å, and c = 8.2093 Å. The comparison data with the experimentally measured lattice constants can be found in Supplementary Materials Table S5. By replacing Ta atoms with metal atoms Cd, Sn, and Hf, the corresponding structure type is acquired, as shown in Figure 1b–d. The relevant data on atomic coordinates and atomic displacement differences before and after optimization in the metal atom-doped KTN system are presented in Tables S1–S3 of Supplementary Materials.

Based on density functional theory, calculations were performed using the VASP package (version 6.0) [22]. The generalized gradient approximation (GGA) with the Perdew–Burke–Ernzerhof (PBE) functional was employed for the exchange–correlation energy [23]. Although we found that in density functional calculations the PBE functional tends to underestimate band gaps, the focus of this paper is on comparing the trend changes in the photocatalytic performance effects of doping KTN crystal systems with elements with similar structures: Cd, Sn, and Hf. This computational characteristic does not affect the trend comparison conclusions. Additionally, the computational cost of PBE is only 1/20–1/50 that of HSE06, with robust convergence properties that enable easy handling of systems containing hundreds of atoms [24,25]. Geometric optimization was conducted for both pristine and doped systems. A 3 × 3 × 3 k-point grid was used, with the self-consistent accuracy set to 1.0 × 10^−6^ eV. All calculations were performed in reciprocal space. The electronic configurations of the atoms involved in this study are as follows: K(4s^1^), Nb(4d^5^s^1^), Ta(5d^3^6s^2^), O(2s^2^2p^4^), Cd(4d^10^5s^2^), Sn(5s^2^5p^2^), and Hf(5d^2^6s^2^).

To ensure the rationality of the calculations, this paper evaluates the stability of KTN crystals before and after doping using tolerance factors and octahedral factors prior to calculating various performance metrics. The formulas for the tolerance factor *τ* and octahedral factor μ are, respectively [26]:

(1) $$\tau =(R_{A}+R_{O})/[\sqrt{2}(R_{A}+R_{O})]$$

(2) $$\mu =(R_{B}/R_{O})$$

Here, *R_A_*, *R_B_*, and *R_O_* represent the effective radius of *A*, *B*, and *O* ions, respectively. In this paper, for the KTN system, *R_A_* = *R_K_*^1+^ = 1.33 Å, *R_B_* = *R_Ta_*^5+^ = 0.64 Å, *R_Nb_*^5+^ = 0.69 Å, and *R_O_* = *R_O_*^2−^ = 1.4 Å. The tolerance factor calculated is 0.935. The tolerance factors of the doping systems are shown in Table 1. The results show that the tolerance factor values of each doping system are similar and basically consistent with those of the original KTN systems, indicating that element doping does not change the stability of the crystal structure.

## 3. Structural Characterization and Bond Length Analysis

Table 2 presents the lattice constants, volumes, energies, binding energies, and bond length of the KTN system before and after doping. In the table, the lattice constants a and b of all doping systems increase compared with the original KTN system. The lattice constant c decreases in KTN-Sn compared to the original KTN but increases in all other systems, indicating that the doping of elements causes lattice distortion. The volume changes in each system show that the volume of KTN increases after doping, primarily because the ion radii of Ta (R_Ta_^5+^ = 0.64) and N (R_Nb_^5+^ = 0.69) are smaller than those of the dopant elements. The bond length represents the average bond length between metal Ta and doped metals Cd, Sn, Hf with O atoms, respectively. Compared with the Ta-O bond length, the Cd-O, Sn-O, and Hf-O bond lengths all increase, further confirming that lattice distortion occurs in the system. This distortion alters the band structure broussonetia papyrifera of the crystal and affects the spontaneous polarization, which can promote electron migration and is particularly beneficial for the dissociation of photogenerated electrons and utetheisa kong hole pairs.

The formation energy is a key parameter for evaluating the thermodynamic stability of materials, and its core formula is as follows:

(3) $$E_{f}=E_{total}-\sum_{i}n_{i}\mu_{i}$$

Here, *E_f_* represents the system’s formation energy, *E_total_* denotes the total energy of the doped system, *n_i_* represents the number of atoms of element *i* in the system, and *μ_i_* represents the chemical potential of element *i.* As shown in the table, the formation energy of the doped system is all negative, so the formation of the compound is an exothermic process, indicating that the doped system can exist stably [27].

The binding energy is a crucial parameter for measuring the strength of atomic bonding, and its calculation formula is as follows:

(4) $$E_{b}=E_{total}-(E_{KTN}+E_{M})$$

Here, *E_b_* represents the system’s binding energy, *E_total_* denotes the total energy of the doped system, *E_KTN_* is the total energy of the pure KTN crystal, and *E_M_* stands for the energy of the isolated metal atom (Cd/Sn/Hf). If the value of *E_b_* is less than 0, it indicates that the system’s energy decreases after doping, and the bonding process is spontaneous and stable [27,28]. In the table, it can be observed that the *E_b_* values of all doping systems are all negative, indicating that the doped system remains stable.

## 4. Electronic Structure

### 4.1. Band Structure Analysis

**Figure 2:**
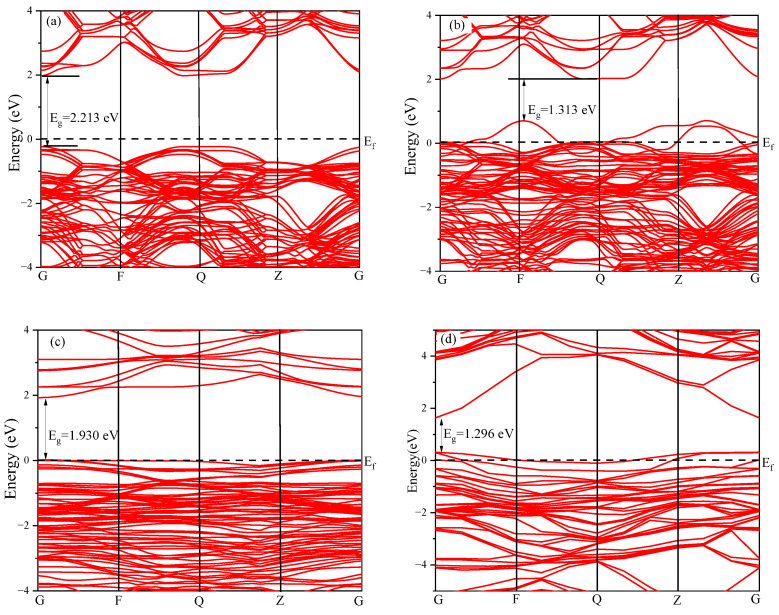
Band diagram of KTN systems before and after doping: (**a**) KTN; (**b**) KTN-Cd; (**c**) KTN-Sn; (**d**) KTN-Hf.

To investigate the effect of doping elements on the bandgap width of the systems, we analyzed the energy levels near the Fermi level (−5 to 5 eV), setting the Fermi level at 0 eV. Figure 2 illustrates the band structure of the KTN systems before and after doping. In Figure 2a, the original KTN system has a bandgap width of 2.213 eV, with the Fermi level near the valence band top. The electrons in this system transition directly from the Γ point to the Γ point, indicating a direct bandgap. The conduction band primarily starts at about 2 eV, while the valence band begins at −0.3 eV. Near the Fermi level, the number of valence bands is significantly higher than that of conduction bands. In Figure 2b, the KTN-Cd system has a reduced bandgap of 1.313 eV, with the conduction band shifting towards lower energy levels. The valence band shifts upward, where the energy level of the valence band is higher than the Fermi level, and there are many holes, presenting the characteristics of p-type semiconductors. In Figure 2c, the KTN-Sn system has a bandgap width of 1.930 eV, which is larger than that of the Cd-doped system but smaller than that of the original KTN system. This system also exhibits the characteristics of a p-type semiconductor. In Figure 2d, the band structure diagram of KTN-Hf shows a bandgap width of 1.296 eV, with its valence band position shifted upward above the Fermi level, while holes act as the majority carriers, exhibiting p-type characteristics. Therefore, the introduction of Cd, Sn, and Hf, whose valence state is lower than that of Ta^5+^, reduces the band gap of the KTN system, induces the formation of a p-type semiconductor, and enhances the carrier mobility of the system, thus improving the photocatalytic performance [29].

In summary, compared with the original KTN system, the bandgap width of the doped systems all decreases. Furthermore, by replacing Ta^5+^ ions with Cd^2+^, Sn^4+^, and Nb^5+^ ions, an excess of holes is introduced, thereby transforming each doped system into a p-type semiconductor. Specifically, the valence bands of the KTN-Cd, KTN-Sn, and KTN-Hf systems shifts upward, with their tops located above the Fermi level. These features collectively and effectively inhibit the recombination of photogenerated carriers and holes, significantly increasing the probability of electron transitions and thereby enhancing the photocatalytic efficiency of the systems.

### 4.2. State Density Analysis

**Figure 3:**
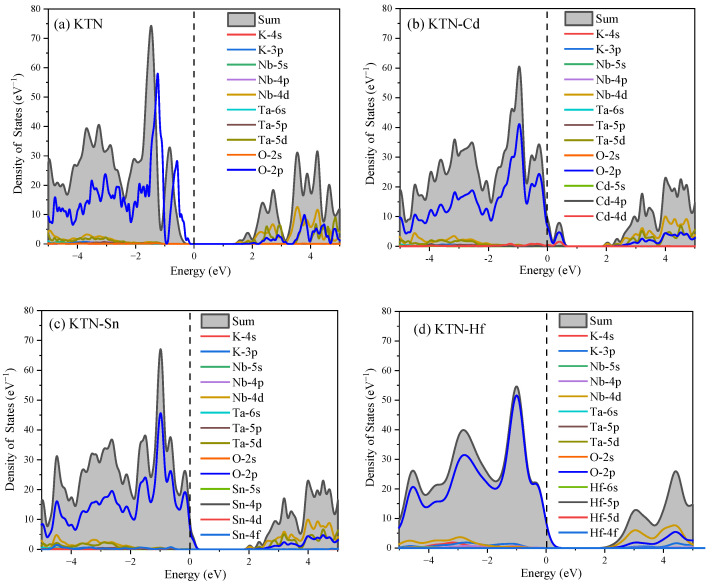
Density of states before and after doping in KTN systems: (**a**) KTN; (**b**) KTN-Cd; (**c**) KTN-Sn; (**d**) KTN-Hf.

To better understand the contributions of atomic orbitals in the research system, this paper analyzes the state density diagrams of the KTN systems before and after doping, as shown in Figure 3. In Figure 3a, the state density diagram of the original KTN system shows that the atomic orbitals of Nb-4d, Ta-5d, and O-2p overlap to contribute to the conduction band; in the valence band, the O-2p atomic orbital is the primary contributor. In Figure 3b, the density of states diagram for the KTN-Cd system indicates that the conduction band arises from the hybridization of Nb-4d, Ta-5d, and O-2p atomic orbitals, whereas the valence band is predominantly composed of the O-2p states. The p-d hybridization between the O-2p and Cd-4d states within the 0~1 eV range causes a peak in the system, providing a shortcut for electron transitions. In Figure 3c, the state density diagram of the KTN-Sn system shows that the conduction band is still primarily contributed to by Nb-4d, Ta-5d, and O-2p states; the valence band is mainly contributed to by the O-2p state; and the O-2p state forms a band tail effect near the Fermi level, consistent with the results of the previous band analysis. In Figure 3d, the state density diagram of the KTN-Hf system shows that the interaction of Nb-4d, Ta-5d, and O-2p primarily contributes to the conduction band; the valence band is mainly contributed to by the O-2p state, which causes the valence band to pass through the Fermi level, resulting in a band tail effect.

In summary, the conduction band of the doping KTN system is primarily contributed to by the interaction of Nb-4d, Ta-5d, and O-2p states. The valence band is mainly formed by the interaction between the O-2p atomic orbitals and the atomic orbitals of the dopant elements. Moreover, the doping system exhibits band tailing and hybridization effects, which significantly reduce the difficulty of electron transitions.

## 5. Optical Properties

### 5.1. Dielectric Function

**Figure 4:**
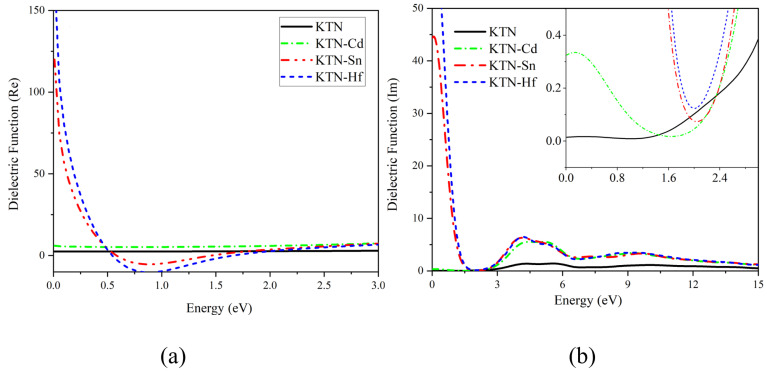
Dielectric function diagram of KTN systems before and after doping: (**a**) real part diagram; (**b**) imaginary part diagram.

Figure 4 illustrates the variations in dielectric function with photon energy for pristine and doped KTN systems. In Figure 4a depicts the real part of the dielectric function. The static dielectric constants (values at photon energy approaching 0 eV) are determined as 2.4734 (pristine), 5.7212 (KTN-Cd), 119.1935 (KTN-Hf), and 174.7538 (KTN-Sn). These results demonstrate significantly enhanced static dielectric constants in all doped systems compared to the pristine sample, with the KTN-Sn system exhibiting the strongest charge screening capacity and polarization capability. The curve displays a local minimum near 0.75 eV, followed by a sharp decline in the 0–0.75 eV energy range. Beyond 0.75 eV, the real component gradually increases and approaches zero at 0.3 eV. These characteristics confirm the transparent conductive thin-film behavior of KTN materials, consistent with findings reported by Yamane et al. [30]. Figure 4b presents the imaginary part, which reveals absorption peaks at 4.285 eV (pristine), 0.148 eV (KTN-Cd), 0.120 eV (KTN-Hf), and 0.068 eV (KTN-Sn). All doped systems exhibit red-shifted absorption peaks toward lower energies. Among them, the KTN-Sn system demonstrates the most pronounced redshift effect and extends its response range into the infrared region. Notably, although this system does not possess the narrowest bandgap, it achieves the broadest response range to the solar spectrum. This further demonstrates that optimizing photocatalytic performance depends critically on attaining an appropriate bandgap width.

### 5.2. Absorption Spectrum

**Figure 5:**
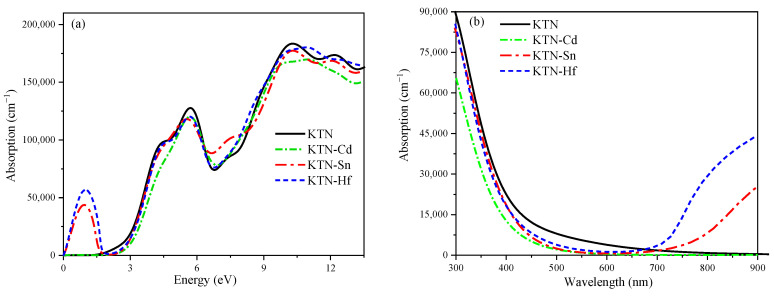
Absorption spectra of KTN before and after doping: (**a**) absorption band edge diagram; (**b**) absorption spectrum.

Figure 5 shows the absorption spectra of the KTN systems before and after doping. The absorption edge diagrams of the KTN systems before and after doping are shown in Figure 5a. The peaks appear at 0.6020, 10.5993, 0.9142, and 0.9973, respectively. This indicates that the doping expands the absorption range of visible light and causes a redshift in the systems. This is mainly due to the high reactivity of Cd, Sn, and Hf [31,32]. Therefore, in the visible light range (1.64~3.19 eV), the doped system is more efficient in utilizing solar energy. In Figure 5b, the optical absorption spectra of the KTN systems in the visible light range before and after doping are shown. It can be observed that the degree of redshift in the systems before and after doping, from highest to lowest, is KTN-Hf, KTN-Sn, and KTN-Cd. Additionally, Sn, Hf, and Cd expand into the far-infrared region. Studies have shown that the polarization intensities of Hf, Cd, and Sn-doped ferroelectric materials are 0.440, 0.052, and 0.0393 C/m^2^, respectively. Thus, doping with these elements accelerates the separation rate of electrons and holes within the systems, thereby expanding the absorption range of solar energy and significantly enhancing photocatalytic activity, which aligns with the optical property changes observed in this study.

## 6. Effective Mass

If you want to study the optical and electrical properties of the system more clearly, then effective mass is one of the effective ways [33]. Therefore, this paper also calculated and analyzed the effective mass of KTN systems before and after doping. The formula of effective mass [34] is as follows:

(5) $$m^{*}=\hbar^{2}/(\frac{\partial^{2}E}{\partial k^{2}})$$

Here, m* is effective mass, and *E* is the energy of an electron or a hole. The unit of $\frac{\partial^{2}E}{\partial k^{2}}$ is 1/(2π/a)^2^ eV. The wave vector is k=2π/λ, with λ representing the de Broglie wavelength and ℏ = h/2π denoting the reduced Planck constant. The E-k data are fitted by the quadratic curve *E*(*k*) = *E*_0_ + d_1_*k* + d_2_*k*^2^, and the effective mass can be written as:

(6) $$m*=\hbar^{2}/(\frac{\partial^{2}E}{\partial k^{2}})={\left(h/2\pi \right)}^{2}/2d_{2}{\left(2\pi /a\right)}^{2}=h^{2}/2d_{2}a^{2}$$

The lattice constant *a* is measured in units of 10^−10^ m. First, the E-k data points are fitted with a polynomial [35,36], and then the fitted curve is numerically second-differentiated rather than the original data. This approach not only reduces the number of required data points but also significantly saves computational time and enhances the accuracy of the fit. The fitting results are as follows: *E* = −16.40835 + 51.97139 × *k* − 41.09499 × *k*^2^, which allows us to derive the result of $(\partial^{2}E)/(\partial k^{2})=2d_{2}$. Substituting this result into Formula (2) yields the effective mass of the KTN systems before and after doping. The calculation results are shown in Table 3 (the rest mass of an electron *m_0_* = 9.11 × 10^−31^ kg). The table shows that the effective mass of holes (m*_h_) is significantly higher than that of electrons (m*_e_). Photogenerated electrons rapidly migrate to the catalyst surface to participate in the reduction reaction, while holes remain at the oxidation sites in the valence band. The system exhibits a heavy hole–light electron configuration.

The D value represents the ratio of the effective mass (m*_h_) of holes to that of electrons (m*_e_), and its equation is expressed as *D* = m*_h_/m*_e_. The value provides a basis for determining whether photogenerated electrons and holes can be utilized in the photocatalytic water splitting process [37]. Compared with original KTN, the D value of the doped systems significantly increases, indicating that the recombination rate of photogenerated electrons and holes in the doped systems decreases, thereby effectively enhancing the photocatalytic efficiency of the material. The D value results indicate that doping of elements such as Cd, Sn, and Hf significantly enhances the KTN system’s ability to inhibit electron–hole pair recombination, thereby promoting its photocatalytic performance. This observation aligns with the analysis results from both the dielectric spectrum and the absorption spectrum. Compared to the original KTN system, doping with Cd, Sn, and Hf elements significantly increases the D value, effectively enhancing the original system’s ability to suppress electron–utetheisa kong hole pair recombination and markedly promoting the dissociation of photogenerated carriers, thereby improving its photocatalytic performance. Meanwhile, the D values of all doped systems are greater than 1, exhibiting heavy hole–light electron characteristics [38]. This phenomenon is highly consistent with the analysis results from dielectric spectroscopy and absorption spectroscopy.

## 7. Conclusions

This study employs first-principles calculations to systematically investigate the effects of transition metal element (Cd, Sn, Hf) doping on the stability, crystal structure, electronic structure, optical properties, and carrier effective mass of original KTN systems. The key findings are summarized as follows:

Doping induces changes in lattice constants and causes lattice distortion. The difference in ionic radii between the dopant atoms (Cd/Sn/Hf) and Nb/Ta atoms leads to an overall increase in crystal volume. However, the calculated total energy and binding energy for all doped systems are negative and comparable, indicating that the doped systems retain good stability. This conclusion is further supported by calculations of the tolerance factor. Analysis of chemical bonds, based on metal–oxygen (M-O) bond lengths, suggests a pronounced covalent character within the systems.

Metal doping significantly reduces the bandgap of the original KTN system and induces either p-type semiconductor characteristic. By doping with Cd, Sn, or Hf, the top of the valence band of the doped system is higher than the Fermi level. However, the band gap still exists in the system, which indicates that the system has become a p-type semiconductor. This modification of the band structure effectively suppresses the recombination of photogenerated electron–hole pairs, thereby enhancing photocatalytic efficiency. Specifically, the conduction band minimum (CBM) is primarily contributed to by Nb-4d, Ta-5d, and O-2p orbitals. The valence band maximum (VBM) originates from the interaction between O-2p orbitals and the atomic orbitals of the dopant metal atoms. Doping also introduces band tailing effects and orbital hybridization phenomena, significantly lowering the energy required for electronic transitions. The chemical activity of the doped metal elements results in a substantial increase in the static dielectric constant compared to original KTN. The absorption spectrum exhibits a redshift, with absorption peaks extending into the infrared and even far-infrared regions. Furthermore, doping enhances the system’s light absorption capacity and photoelectric conversion efficiency, consequently improving electrical conductivity.

Calculations reveal that the effective mass of holes is greater than that of electrons in all doped systems, characterizing them as “heavy-hole, light-electron” systems. This property facilitates the spatial separation of photogenerated electrons and holes, providing further evidence that metal doping significantly enhances the photocatalytic activity of the system.

In summary, transition metal (Cd/Sn/Hf) doping significantly enhances the photocatalytic activity of original KTN materials by reducing the band gap of the original KTN system, inducing p-type semiconductor properties, enhancing the light absorption and dielectric constant, promoting absorption redshift, and forming “heavy-hole, light-electron” carrier separation characteristics.

## Figures and Tables

**Table 1:** The effective ionic radius of each doped atom and the tolerance factors of the corresponding doping systems.

Parameters	Cd	Sn	Hf
Ionic radius/Å	0.97	0.69	0.71
Tolerance factor	0.892	0.935	0.931
Octahedral factor	0.69	0.49	0.50

**Table 2:** Lattice constants, volumes, and energy values of the KTN systems before and after doping.

System	a/Å	b/Å	c/Å	V/Å^3^	Bond Length/Å	E_total_/eV	E_f_	E_b_
KTN	8.0187	8.0187	8.1753	530.0340	2.02 (Ta-O)	–14348.5	–7.9287	–7.9287
KTN-Cd	8.0947	8.1898	8.1925	543.1075	2.17 (Cd-O)	–18493.1	–0.0076	–7.6840
KTN-Sn	8.0480	8.1233	8.1422	532.3017	2.09 (Sn-O)	–11565.3	–0.1181	–7.5970
KTN-Hf	8.0682	8.0988	8.1822	534.6442	2.09 (Hf-O)	–18838.8	–0.0149	–7.8761

**Table 3:** Effective mass of electrons and holes in KTN systems before and after doping.

System	KTN	Cd-KTN	Sn-KTN	Hf-KTN
m*_e_/10^−31^ kg	0.4584	0.0993	0.1033	0.1003
m*_h_/10^−31^ kg	0.1905	18.7428	5.9030	8.1096
D(m*_h_/m*_e_)/10^−31^ kg	0.4156	188.7492	57.1442	80.8534

## Data Availability

The original contributions presented in this study are included in the article, and further inquiries can be directed to the corresponding authors.

## Supplementary Materials

The following supporting information can be downloaded at: https://www.mdpi.com/article/10.3390/nano15171322/s1, Table S1: Cd-KTN system’s atomic coordinates and atomic displacement differences before and after optimization; Table S2: Sn-KTN system’s atomic coordinates and atomic displacement differences before and after optimization; Table S3: Hf-KTN system#x2019;s atomic coordinates and atomic displacement differences before and after optimization; Table S4: Comparison of spontaneous polarization coefficients (Ps) and Curie temperatures (Tc) among KTN, KNN, and NN systems; Table S5; Comparison of experimental measurement and theoretical calculation of lattice constants in KTN systems. References [11,13,39,40,41] are cited in the supplementary materials.
