# Supplementary material for: First-Principles Study on the Photocatalytic Performance of K(Ta0.5Nb0.5)O3 Doped with Metals (Cd, Sn, Hf)
Source: Nanomaterials (Basel). 2025 Aug 28;15(17):1322. doi: 10.3390/nano15171322 (PMC12430202; doi:10.3390/nano15171322)
Supplement: Supplementary file 1 [file nanomaterials-15-01322-s001.zip › nanomaterials-3783706-supplementary.pdf]

First-Principles Study on the Photocatalytic Performance of $K(Ta_{0.5}Nb_{0.5})O_3$ Doped with Metals (Cd, Sn, Hf)

Can Zhao [1], Qiao-Yue Chen [1], Xin-Yuan Zhou [1], Xu-Cai Zhao [1], Bo-Cheng Lei [1], and Li-Li Zhang [1,*], Zhao Jing [2], Yi-Neng Huang [1,3,*]

[1] Xinjiang Laboratory of Phase Transitions and Microstructures in Condensed Matters, College of Physical Science and Technology, Yili Normal University, Yining 835000, China;

[2] Xinjiang Key Laboratory of Clean Conversion and High Value Utilization of Biomass Resources; School of Chemistry and Chemical Engineering, Yili Normal University, Yining 835000, China;

[3] National Laboratory of Solid State Microstructures, School of Physics, Nanjing University, Nanjing 210093, China.

* Correspondence: zhanglili@ylnu.edu.cn (L.Z.); ynhuang@nju.edu.cn (Y.N.)

Tables S1-S3 present the atomic coordinates and relative displacements of the Cd-KTN, Sn-KTN, and Hf-KTN systems before and after optimization, where x, y, z denote the coordinate points before optimization, x', y', z' denote the coordinate points after optimization, and Δx, Δy, Δz correspond to the relative displacement magnitudes in the x, y, and z directions, respectively.

Table S1. Cd-KTN system's atomic coordinates and atomic displacement differences before and after optimization

Atom	x	y	z	x′	y′	z′	Δx	Δy	Δz
Nb1	0.25003	0.24205	0.24964	0.25013	0.23984	0.25513	0.0001	-0.00221	0.00549
Nb2	0.75003	0.23982	0.25615	0.75013	0.23663	0.25354	0.0001	-0.00319	-0.00261
Nb3	0.25002	0.74204	0.24959	0.25004	0.73727	0.26545	0.00002	-0.00477	0.01586
Nb4	0.75003	0.73982	0.25617	0.75003	0.73551	0.25691	0	-0.00431	0.00074
O1	0.25000	0.25183	0.2020	0.24998	0.25047	0.2061	-0.00002	-0.00136	0.0041
O2	0.24999	0.0363	0.26805	0.24998	0.00961	0.26337	-0.0001	-0.02669	-0.00468
O3	0.00061	0.25399	0.26823	-0.00063	0.25435	0.26788	-0.00124	0.00036	-0.00035
O4	0.74999	0.25236	0.2484	0.74998	0.25259	0.2189	-0.00001	0.00023	-0.0295
O5	0.74999	0.00334	0.26919	0.74998	0.00844	0.26886	-0.00001	0.0051	-0.00033
O6	0.49937	0.25399	0.26822	0.50049	0.25435	0.26788	0.00112	0.00036	-0.00034
O7	0.25000	0.75182	0.2025	0.24999	0.75243	0.03367	-0.00001	0.00061	-0.16883
O8	0.24999	0.50365	0.26805	0.24998	0.50224	0.26367	-0.00001	-0.00141	-0.00438
O9	0.00062	0.75399	0.26823	-0.00366	0.75396	0.26491	-0.00428	-0.00003	-0.00332
O10	0.74999	0.75236	0.2480	0.74999	0.75203	0.02170	0	-0.00033	-0.2263
O11	0.74999	0.50334	0.26919	0.74998	0.50648	0.26895	-0.00001	0.00314	-0.00024
O12	0.49936	0.75399	0.26823	0.50361	0.75396	0.26491	0.00425	-0.00003	-0.00332
O13	0.25000	0.25176	0.51839	0.24999	0.25069	0.51609	-0.00001	-0.00107	-0.0023
O14	0.25000	0.00295	0.76687	0.25000	0.01680	0.76524	0	0.01385	-0.00163
O15	0.00691	0.25439	0.77086	-0.00258	0.24998	0.76854	-0.00949	-0.00441	-0.00232
O16	0.74999	0.25275	0.51178	0.74998	0.25302	0.52178	-0.00001	0.00027	0.01
O17	0.75000	0.00199	0.77970	0.75000	0.00440	0.76864	0	0.00241	-0.01106

O18	0.49309	0.25439	0.77087	0.50257	0.24998	0.76854	0.00948	-0.00441	-0.00233
O19	0.25000	0.75176	0.51835	0.24999	0.75205	0.49583	-0.00001	0.00029	-0.02252
O20	0.25000	0.50295	0.76687	0.25000	0.48456	0.76495	0	-0.01839	-0.00192
O21	0.00688	0.75438	0.77086	-0.01413	0.75119	0.76708	-0.02101	-0.00319	-0.00378
O22	0.75000	0.75275	0.51180	0.75000	0.75237	0.51772	0	-0.00038	0.00592
O23	0.75000	0.50207	0.77970	0.75000	0.49904	0.76867	0	-0.00303	-0.01103
O24	0.49312	0.75438	0.77086	0.51413	0.75119	0.76708	0.02101	-0.00319	-0.00378
K1	0.00305	0.99744	0.01233	0.00345	0.99468	0.00949	0.0004	-0.00276	-0.00284
K2	0.49695	0.99744	0.01233	0.49657	0.99468	0.00949	-0.00038	-0.00276	-0.00284
K3	0.00305	0.49744	0.01233	0.00362	0.50059	0.00910	0.00057	0.00315	-0.00323
K4	0.49695	0.49744	0.01233	0.49640	0.50059	0.00910	-0.00055	0.00315	-0.00323
K5	0.00261	0.99747	0.50537	0.00308	0.99516	0.51221	0.00047	-0.00231	0.00684
K6	0.49740	0.99747	0.50537	0.49693	0.99516	0.51221	-0.00047	-0.00231	0.00684
K7	0.00261	0.49746	0.50536	0.00306	0.50093	0.51296	0.00045	0.00347	0.0076
K8	0.49739	0.49746	0.50536	0.49696	0.50093	0.51296	-0.00043	0.00347	0.0076
Ta1	0.24999	0.23988	0.74858	0.25000	0.25053	0.75488	0.00001	0.01065	0.0063
Ta2	0.75001	0.25102	0.75706	0.75000	0.24992	0.75359	-0.00001	-0.0011	-0.00347
Ta3	0.75000	0.75108	0.75704	0.75000	0.74986	0.75695	0	-0.00122	-0.00009
Cd1	0.25000	0.73987	0.74862	0.25000	0.75158	0.76559	0	0.01171	0.01697

Table S2. Sn-KTN system's atomic coordinates and atomic displacement differences before and after optimization

Atom	x	y	z	x′	y′	z′	**Δ**x	**Δ**y	**Δ**z
Nb1	0.25008	0.23931	0.25446	0.25008	0.23931	0.25446	0	0	0
Nb2	0.75008	0.23809	0.25448	0.75008	0.23809	0.25448	0	0	0
Nb3	0.25005	0.73833	0.26422	0.25005	0.73833	0.26422	0	0	0
Nb4	0.75005	0.73768	0.25517	0.75005	0.73768	0.25517	0	0	0
O1	0.24999	0.25051	0.01936	0.24999	0.25051	0.01936	0	0	0
O2	0.24998	0.00738	0.26366	0.24998	0.00738	0.26366	0	0	0
O3	-0.00026	0.25411	0.26774	-0.00026	0.25411	0.26774	0	0	0
O4	0.74999	0.25231	0.02165	0.74999	0.25231	0.02165	0	0	0
O5	0.74998	0.00700	0.26906	0.74998	0.00700	0.26906	0	0	0
O6	0.50018	0.25411	0.26774	0.50018	0.25411	0.26774	0	0	0
O7	0.24999	0.75121	0.02487	0.24999	0.75121	0.02487	0	0	0
O8	0.24998	0.50350	0.26383	0.24998	0.50350	0.26383	0	0	0
O9	-0.00190	0.75392	0.26531	-0.00190	0.75392	0.26531	0	0	0
O10	0.74999	0.75208	0.02156	0.74999	0.75208	0.02156	0	0	0
O11	0.74998	0.50576	0.26911	0.74998	0.50576	0.26911	0	0	0
O12	0.50184	0.75392	0.26531	0.50184	0.75392	0.26531	0	0	0
O13	0.24999	0.25060	0.51645	0.24999	0.25060	0.51645	0	0	0
O14	0.25000	0.00864	0.76527	0.25000	0.00864	0.76527	0	0	0
O15	-0.00135	0.25056	0.76860	-0.00135	0.25056	0.76860	0	0	0

O16	0.74999	0.25269	0.52090	0.74999	0.25269	0.52090	0	0	0
O17	0.75000	0.00361	0.77007	0.75000	0.00361	0.77007	0	0	0
O18	0.50134	0.25056	0.76861	0.50134	0.25056	0.76861	0	0	0
O19	0.24999	0.75130	0.50635	0.24999	0.75130	0.50635	0	0	0
O20	0.25000	0.49335	0.76527	0.25000	0.49335	0.76527	0	0	0
O21	-0.00707	0.75132	0.76749	-0.00707	0.75132	0.76749	0	0	0
O22	0.74999	0.75240	0.51850	0.74999	0.75240	0.51850	0	0	0
O23	0.75000	0.5046	0.77005	0.75000	0.50046	0.77005	0	-0.00414	0
O24	0.50706	0.75132	0.76749	0.50706	0.75132	0.76749	0	0	0
K1	0.00134	0.99671	0.01110	0.00134	0.99671	0.01110	0	0	0
K2	0.49867	0.99671	0.01110	0.49867	0.99671	0.01110	0	0	0
K3	0.00160	0.49867	0.01087	0.00160	0.49867	0.01087	0	0	0
K4	0.49841	0.49867	0.01087	0.49841	0.49867	0.01087	0	0	0
K5	0.00123	0.99697	0.50971	0.01230	0.99697	0.50971	0.01107	0	0
K6	0.49878	0.99697	0.50971	0.49878	0.99697	0.50971	0	0	0
K7	0.00128	0.49912	0.51021	0.01280	0.49912	0.51021	0.01152	0	0
K8	0.49873	0.49912	0.51021	0.49873	0.49912	0.51021	0	0	0
Ta1	0.25000	0.25080	0.75418	0.25000	0.25080	0.75418	0	0	0
Ta2	0.75000	0.24962	0.75582	0.75000	0.24962	0.75582	0	0	0
Ta3	0.75000	0.74945	0.75627	0.75000	0.74945	0.75627	0	0	0
Sn1	0.25000	0.75119	0.76537	0.25000	0.75119	0.76537	0	0	0

Table S3. Hf-KTN system's atomic coordinates and atomic displacement differences before and after optimization

Atom	x	y	z	x′	y′	z′	Δx	Δy	Δz
Nb1	0.25005	0.24882	0.25132	0.25005	0.24882	0.25132	0	0	0
Nb2	0.75005	0.23938	0.25302	0.75005	0.23938	0.25302	0	0	0
Nb3	0.75000	0.73793	0.25306	0.75000	0.73793	0.25306	0	0	0
Nb4	0.24999	0.25021	0.01698	0.24999	0.25021	0.01698	0	0	0
O1	0.24999	0.00910	0.26666	0.24999	0.00910	0.26666	0	0	0
O2	-0.00113	0.25197	0.26735	-0.00113	0.25197	0.26735	0	0	0
O3	0.74999	0.25198	0.02162	0.74999	0.25198	0.02162	0	0	0
O4	0.74999	0.00668	0.26870	0.74999	0.00668	0.26870	0	0	0
O5	0.50108	0.25197	0.26734	0.50108	0.25197	0.26734	0	0	0
O6	0.25000	0.75095	0.00797	0.25000	0.75095	0.00797	0	0	0
O7	0.24999	0.49308	0.26675	0.24999	0.49308	0.26675	0	0	0
O8	-0.00754	0.75385	0.26796	-0.00754	0.75385	0.26796	0	0	0
O9	0.75000	0.75159	0.01950	0.75000	0.75159	0.01950	0	0	0
O10	0.74999	0.50351	0.26861	0.74999	0.50351	0.26861	0	0	0
O11	0.50754	0.75385	0.26797	0.50754	0.75385	0.26797	0	0	0
O12	0.25000	0.25005	0.52053	0.25000	0.25005	0.52053	0	0	0
O13	0.25000	0.00294	0.76470	0.25000	0.00294	0.76470	0	0	0

O14	0.00037	0.25264	0.6912	0.00037	0.25264	0.76912	0	0	0.07792
O15	0.74999	0.25235	0.52414	0.74999	0.25235	0.52414	0	0	0
O16	0.75000	0.00348	0.77223	0.75000	0.00348	0.77223	0	0	0
O17	0.49962	0.25264	0.6913	0.49992	0.25264	0.76913	0.0003	0	0.07783
O18	0.25000	0.75088	0.52257	0.25000	0.75088	0.52257	0	0	0
O19	0.25000	0.50063	0.76478	0.25000	0.50063	0.76478	0	0	0
O20	-0.00058	0.75245	0.76702	-0.00058	0.75245	0.76702	0	0	0
O21	0.75000	0.75207	0.52441	0.75000	0.75207	0.52441	0	0	0
O22	0.75000	0.50279	0.77227	0.75000	0.50279	0.77227	0	0	0
O23	0.50058	0.75245	0.6701	0.50058	0.75245	0.76701	0	0	0.09691
O24	0.00152	0.99731	0.01197	0.00152	0.99731	0.01197	0	0	0
K1	0.49848	0.99731	0.01197	0.49848	0.99731	0.01197	0	0	0
K2	0.00159	0.49805	0.01207	0.00159	0.49805	0.01207	0	0	0
K3	0.49841	0.49806	0.01207	0.49841	0.49806	0.01207	0	0	0
K4	0.00084	0.99777	0.50740	0.00084	0.99777	0.50740	0	0	0
K5	0.49917	0.99777	0.50740	0.49917	0.99777	0.50740	0	0	0
K6	0.00098	0.49783	0.50732	0.00098	0.49783	0.50732	0	0	0
K7	0.49903	0.49783	0.50732	0.49903	0.49783	0.50732	0	0	0
K8	0.25005	0.24882	0.25132	0.25005	0.24882	0.25132	0	0	0
Ta1	0.25000	0.24490	0.75335	0.25000	0.24490	0.75335	0	0	0
Ta2	0.75000	0.24973	0.75527	0.75000	0.24973	0.75527	0	0	0
Ta3	0.25000	0.74439	0.76151	0.25000	0.74439	0.76151	0	0	0
Hf1	0.25000	0.74926	0.26194	0.25000	0.74926	0.26194	0	0	0

Table S4 presents a comparative data set of spontaneous polarization coefficients and Curie temperature values for KTN, KNN, and NN systems. The results indicate that KNN exhibits the strongest spontaneous polarization capability, while the KTN system demonstrates the highest thermodynamic stability. The data refer to the literature in [11, 13, 39, 40].

Table S4. Comparison of spontaneous polarization coefficients (P_s) and Curie temperatures (T_c) among KTN, KNN, and NN systems

System	P_s/C•m^{-2}	T_c/K
KTN	0.17	313
KNN	0.30	420
NN	0.25	638

Table S5 presents a comparison between the experimentally measured and theoretically calculated lattice constants of the KTN system, where KTN' represents the theoretically calculated system in this study. The experimental value of the KTN lattice constant is referenced in literature [41].

Table S5. Comparison of experimental measurement and theoretical calculation of lattice constants in KTN systems

System	a/Å	b/Å	c/Å
KTN	7.9860	7.9860	7.9860

**KTN'**	**8.0187**	**8.0187**	**8.1753**

References

[11] Li, C.; Wang, X.; Wu, Y.; Liang, F.; Wang, F.; Zhao, X.; Zhang, H. Three-dimensional nonlinear photonic crystal in naturally grown potassium–tantalate–niobate perovskite ferroelectrics. *Light Sci. Appl.* **2020**, *9*, 8.

[13] Huan, Y.; Shen, H.; Zhu, Y.; Li, M.; Li, H.; Wang, Z.; Wei, T. Enhanced ferro-photocatalytic performance for $ANbO_3$ (A = Na, K) nanoparticles. *Math. Biosci. Eng.* **2019**, *16*, 4122–4134.

[39] Peng Y. T.; Tan Z.; An J.; Zhu J. G.; Zhang Q. M. The tunable ferroelectricity and piezoelectricity of the KNN piezoceramics by Na concentrations: First-principles calculations - ScienceDirect. *J Eur Ceram Soc.* **2019**. *39*(16): 5252-5259.

[40] Deng Y. F.; Wang J. J.; Zhang C. X.; Ma H.; Bai C. G.; Liu D. Q.; Wu F. M.; Yang B. Structural and Electric Properties of MnO_2-Doped KNN-LT Lead-Free Piezoelectric Ceramics, *Materials.* **2020**, *10*(8): 705

[41] Wang X. P. , J. Y. Wang, Y. G. Yu, H. J. Zhang and R. I. Boughton. Growth of cubic $KTa_{1-x}Nb_xO_3$ crystal by czochralski method. *J Cryst Growth.* **2006**, *293*(2): 398-403
